# An account of solvent accessibility in protein-RNA recognition

**DOI:** 10.1038/s41598-018-28373-2

**Published:** 2018-07-12

**Authors:** Sunandan Mukherjee, Ranjit Prasad Bahadur

**Affiliations:** Computational Structural Biology Laboratory, Department of Biotechnology, Indian Institute of Technology Kharagpur, Kharagpur, 721302 India

## Abstract

Protein–RNA recognition often induces conformational changes in binding partners. Consequently, the solvent accessible surface area (SASA) buried in contact estimated from the co-crystal structures may differ from that calculated using their unbound forms. To evaluate the change in accessibility upon binding, we compare SASA of 126 protein-RNA complexes between bound and unbound forms. We observe, in majority of cases the interface of both the binding partners gain accessibility upon binding, which is often associated with either large domain movements or secondary structural transitions in RNA-binding proteins (RBPs), and binding-induced conformational changes in RNAs. At the non-interface region, majority of RNAs lose accessibility upon binding, however, no such preference is observed for RBPs. Side chains of RBPs have major contribution in change in accessibility. In case of flexible binding, we find a moderate correlation between the binding free energy and change in accessibility at the interface. Finally, we introduce a parameter, the ratio of gain to loss of accessibility upon binding, which can be used to identify the native solution among the flexible docking models. Our findings provide fundamental insights into the relationship between flexibility and solvent accessibility, and advance our understanding on binding induced folding in protein-RNA recognition.

## Introduction

Protein-RNA recognition is essential for gene expression and its regulations. The initial contact between a RNA-binding protein (RBP) and a RNA, often termed as encounter complex, triggers subsequent conformational changes in order to form a stable and functional association^1,2^. These conformational changes can either be of small scale including side chain movements of amino acid residues or base flipping of nucleotides, or be of large scale movements such as reorientation of polypeptide domains or change in secondary structures of RNA. Moreover, secondary structural transitions can also induce major conformational changes in both the binding partners^3^. It has been observed that the conformational changes upon binding are often associated with significant changes in solvent accessibility in the binding partners. Lee and Richard, in 1971^4^, first coined the term “accessible surface area” to quantify the area of protein surface. Later, Chothia^5,6^ described the correlation between accessible surface area and hydrophobic energy in protein folding. According to his study, the gain in ΔG per squared Angstrom decrease in solvent accessible surface area (SASA) of proteins is 25 cal/mol. Recent studies have shown that relative solvent accessibility can be used to predict the extent of conformational changes associated with protein-protein recognition^7^. Besides, it has also been found that the bound conformations of macromolecules have larger SASA than their unbound states^8^. Moreover, the intrinsic flexibility of proteins can also be measured by their buried and accessible surface area^9^. Recently, Barik *et al*.^10^ showed that the change in SASA upon binding can be used as a parameter to predict the binding hotspots at protein–RNA interfaces. The prerequisite to study the change in solvent accessibility upon protein-RNA binding is the atomic structures of the complexes and their corresponding unbound forms of the binding partners. The growing interests to decipher the 3-dimensional structures of protein-RNA complexes and their unbound structures facilitated the development of protein-RNA docking benchmarks^11–13^.

In this study, we evaluate the change in SASA values calculated from the bound complex and their corresponding unbound components of protein-RNA complexes taken from the docking benchmark version 2^13^. We find, in majority of the cases the interface of both the binding partners gain accessibility in order to provide more surface area to promote the stable interactions. However, majority of RNA non-interface region lose accessibility, while, no such significant bias is observed at the non-interface region of RBPs. The change in interface accessibility is significantly contributed by the side chains, however, a moderate correlation between the change in accessibility and the backbone conformation is also observed. Interestingly, large change in accessibility is observed when the binding is more flexible including large domain movements and secondary structural transition of RBPs upon binding. We find a moderate correlation between the change in accessibility and binding free energy when the interface undergoes significant change in conformation upon binding. Analysis of secondary structural elements reveals that loop-to-helix and helix-to-loop transitions upon binding gain significant accessibility at the interface. Additionally, we show the amino acid residues and nucleotides that do not participate in intermolecular hydrogen bonds (H-bonds) undergo significant change in accessibility upon binding compared to those which are involved in such interactions. We have also investigated the preference of amino acid residues and nucleotides to lose accessibility (buried) or gain accessibility (exposed) upon binding. Finally, we introduce a new parameter, the ratio of gain to loss of solvent accessibility upon binding (GL ratio), which can be effectively used to score the flexible docking solutions to identify the near native structure. These findings have significant applications in designing flexible protein-RNA docking algorithms and engineering protein-RNA interfaces.

## Results

### Dataset of bound and unbound structures

The dataset consists of 126 protein-RNA complexes for which at least one interacting partner is available in the unbound form. Of these 126 complexes, 28 are in class A, 5 are in class B, 40 are in class C and 53 are in class D (refer to Materials and Methods section and Supplementary Table S1). Based on their availability in the unbound form, we find 21 are of P_U_R_U_ type, where both the protein and the RNA are available in the unbound form, 95 are of P_U_R_B_ type, where only the protein is available in the unbound form, and 10 are of P_B_R_U_ type, where only the RNA is available in the unbound form (Table 1). Local alignment of the polypeptide chains between the unbound and the bound (U/B) structures reveals that 93 out of 116 have sequence identity >98%, while the rest have values between 90% and 98%. On the other hand, sequence identity of 20 out of 31 U/B pairs of polynucleotides have values >98% and the rest have values between 90% and 98%. We have discarded 896 (~2%) residues and 96 (~6.7%) nucleotides in the entire dataset due to the mismatch in the alignment between U/B pairs.

**Table 1:** Statistics on change in accessibility upon binding in protein–RNA complexes.

	tRNA (A)	rRNA (B)	duplex RNA (C)	single-stranded RNA (D)	All
No of complexes	28	5	40	53	126
P_U_R_U_	10	3	4	4	21
P_U_R_B_	16	2	32	45	95
P_B_R_U_	2	0	4	4	10
Average B (Å^2^)					
B^B^	2625	1733	2329	1891	2187
Average ASA (Å^2^)					
${B}_{P}^{B}$	1259 (±566)	839 (±276)	1153 (±681)	863 (±580)	1040 (±626)
${A}_{P}^{B}$	23783 (±11457)	8282 (±2342)	18598 (±11530)	16912 (±11836)	18559 (±11903)
${B}_{R}^{B}$	1375 (±736)	965 (±198)	1007 (±284)	1048 (±604)	1132 (±572)
${A}_{R}^{B}$	8028 (±3363)	6553 (±295)	7701 (±6721)	4786 (±4042)	6943 (±4922)
Average δA_P_ (Å^2^)^a^					
Interface	−132.4 (±174.3)	37.5 (±62.8)	−145.9 (±213.7)	−111.5 (±192.1)	−120.5 (±195.2)
Exposed	−174.3 (±167.9)	−23.1 (±20.9)	−167.3 (±209.4)	−183.4 (±169.5)	−172.0 (±184.3)
Buried	43.5 (±35.5)	77.8 (±46.9)	88.8 (±74.0)	87.4 (±75.6)	77.2 (±68.0)
Non-interface	4.5 (±35.3)	33.3 (±20.7)	−10.3 (±31.5)	9.8 (±46.6)	3.4 (±40.1)
Exposed	−25.2 (±16.3)	−5.5 (±NA)	−31.0 (±19.1)	−19.3 (±27.5)	−24.6 (±24.1)
Buried	23.0 (±27.7)	59.2 (±16.7)	26.5 (±24.1)	33.5 (±49.0)	30.4 (±33.5)
Average δA_R_ (Å^2^)^b^					
Interface	−141.0 (±104.3)	−73.9 (±195.2)	16.1 (±135.9)	−174.6 (±67.0)	−92.5 (±143.7)
Exposed	−158.9 (±94.3)	−184.4 (±143.3)	−68.0 (±32.1)	−174.6 (±67.0)	−144.1 (±91.8)
Buried	20.0 (NA)	146.9 (NA)	142.3 (±134.6)	0.0 (NA)	122.7 (±119.1)
Non-interface	16.5 (±40.1)	32.6 (±18.3)	77.9 (±112.2)	26.1 (±41.5)	40.3 (±75.8)
Exposed	−29.7 (±34.0)	0.0 (±NA)	−20.3 (±12.2)	−30.2 (±6.1)	−25.6 (±21.9)
Buried	36.3 (±22.3)	32.6 (±18.3)	143.3 (±100.8)	44.8 (±29.9)	67.3 (±73.5)

^a^Values are calculated on 116 P_U_R_U_ and P_U_R_B_ complexes.

^b^Values are calculated on 31 P_U_R_U_ and P_B_R_U_ complexes.

Standard deviations are provided in parentheses.

### Change in accessibility at the protein-RNA interfaces

The overall change in accessibility upon binding is a cumulative effect of many local conformational rearrangements. Some residues get exposed by burying the others or vice-versa. Change in accessibility of the interface atoms upon binding was calculated by comparing their SASA values in bound and unbound states. On an average, RBPs gain 120.5 Å^2^ of solvent accessibility at the interface upon binding with RNA (Table 1). We find in 92 out of 116 cases, interface region of RBPs gain accessibility upon binding with an average $\delta {A}_{P}^{\mathrm{int}}$ (refer to Materials and Methods section) of −172.0 Å^2^. In the remaining 24 cases, positive changes in $\delta {A}_{P}^{\mathrm{int}}$ are observed with an average of 77.2 Å^2^, indicating a loss in accessibility at the interface. On an average, interface region of RNAs gain 92.5 Å^2^ of solvent accessibility upon binding with RBPs. Majority of them, 80% (25 out of 31), show negative $\delta {A}_{R}^{\mathrm{int}}$ with an average of −144.1 Å^2^ (Table 1). Remaining, only 20%, show positive changes with an average of 122.7 Å^2^, indicating a loss of accessibility.

**Figure 1:**
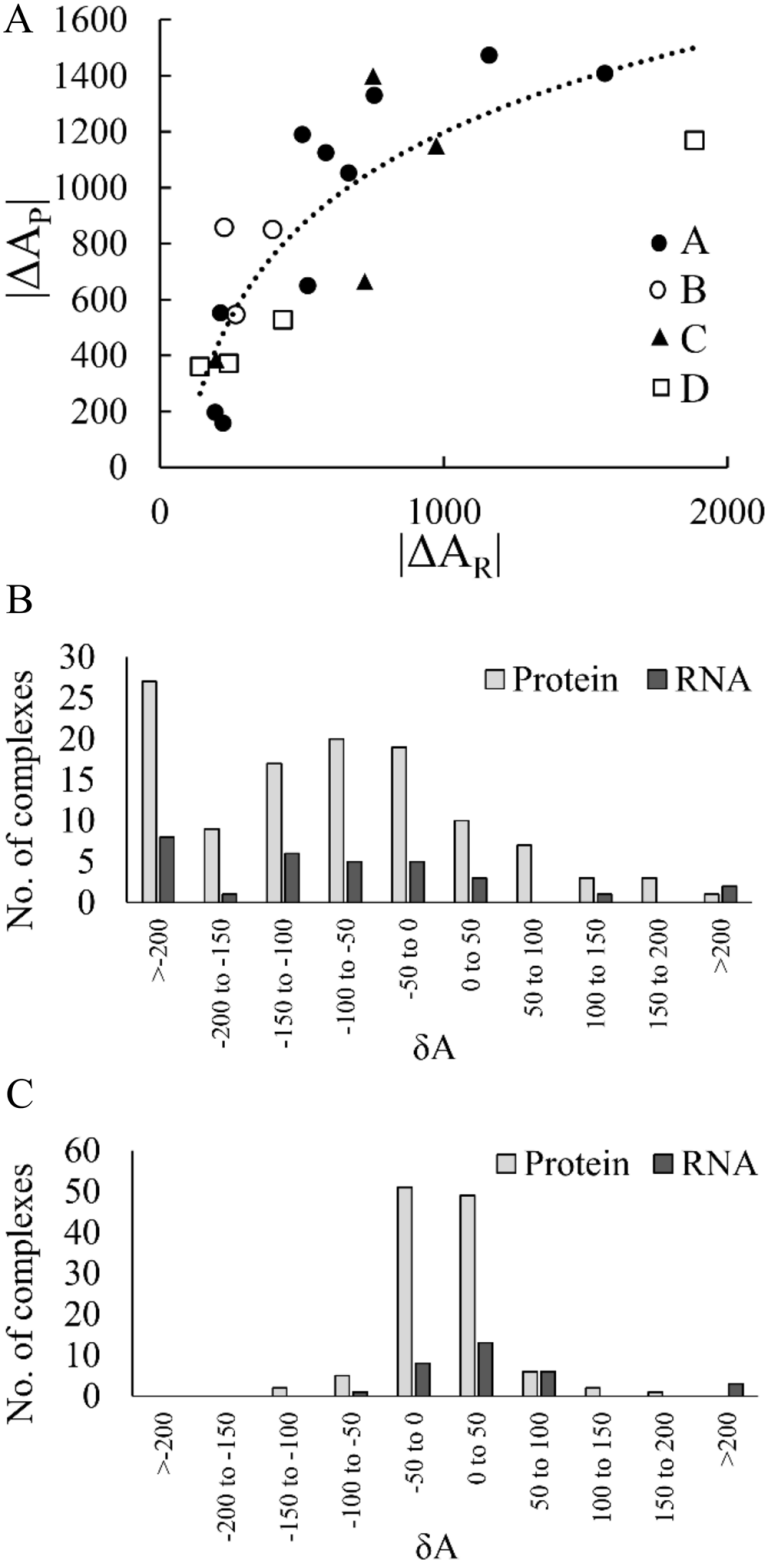
Distribution of change in accessibility in RBPs and RNAs upon binding. **(A)** Correlation between |∆A_P_| and |∆A_R_| at the protein-RNA interfaces for 21 UU cases. The different classes of complexes are shown in different symbols. Distributions of δA in 116 RBPs and in 31 RNAs at the protein-RNA **(B)** interface and **(C)** non-interface regions.

We used 21 P_U_R_U_ cases to quantify the overall change in the accessibility at the interface when both RBPs and their partner RNAs are available in the unbound form. Here, we find an average $\delta {A}_{P+R}^{\mathrm{int}}$ (refer to Materials and Methods section) of −221.2 Å^2^. In this subset, only four complexes lose accessibility upon binding (average $\delta {A}_{P+R}^{\mathrm{int}}$ = 39.2 Å^2^, range is from 22.2 Å^2^ to 59.5 Å^2^), of which two are from class B and one each from class A and class D. In rest of the 17 cases, the change is negative with an average $\delta {A}_{P+R}^{\mathrm{int}}$ of −282.5 Å^2^ (range is from −14.3 Å^2^ to −1285 Å^2^). A significant correlation is observed between ${\rm{\Delta }}{A}_{P}^{\mathrm{int}}$ and ${\rm{\Delta }}{A}_{R}^{\mathrm{int}}$ (R^2^ = 0.7) in the entire dataset (Fig. 1A).

### Change in accessibility at the non-interface region

We have estimated the change in accessibility of amino acid residues and nucleotides at the non-interface region. Here, the average change in accessibility of RBPs is only 3.4 Å^2^ (Table 1), which is significantly lower than that of the interface region. In the entire dataset, 50% RBPs show negative changes with an average of −24.6 Å^2^, and 50% show positive changes with an average of 30.4 Å^2^. At the non-interface region of the RNA, the average change in accessibility is 40.3 Å^2^. In the entire dataset, majority (71%) of the RNAs lose accessibility upon binding with an average $\delta {A}_{R}^{non{\textstyle  \mbox{-} }{\rm{i}}{\rm{n}}{\rm{t}}}$ of 67.3 Å^2^. Only nine RNAs (29%) show negative changes with an average of −25.6 Å^2^.

**Figure 2:**
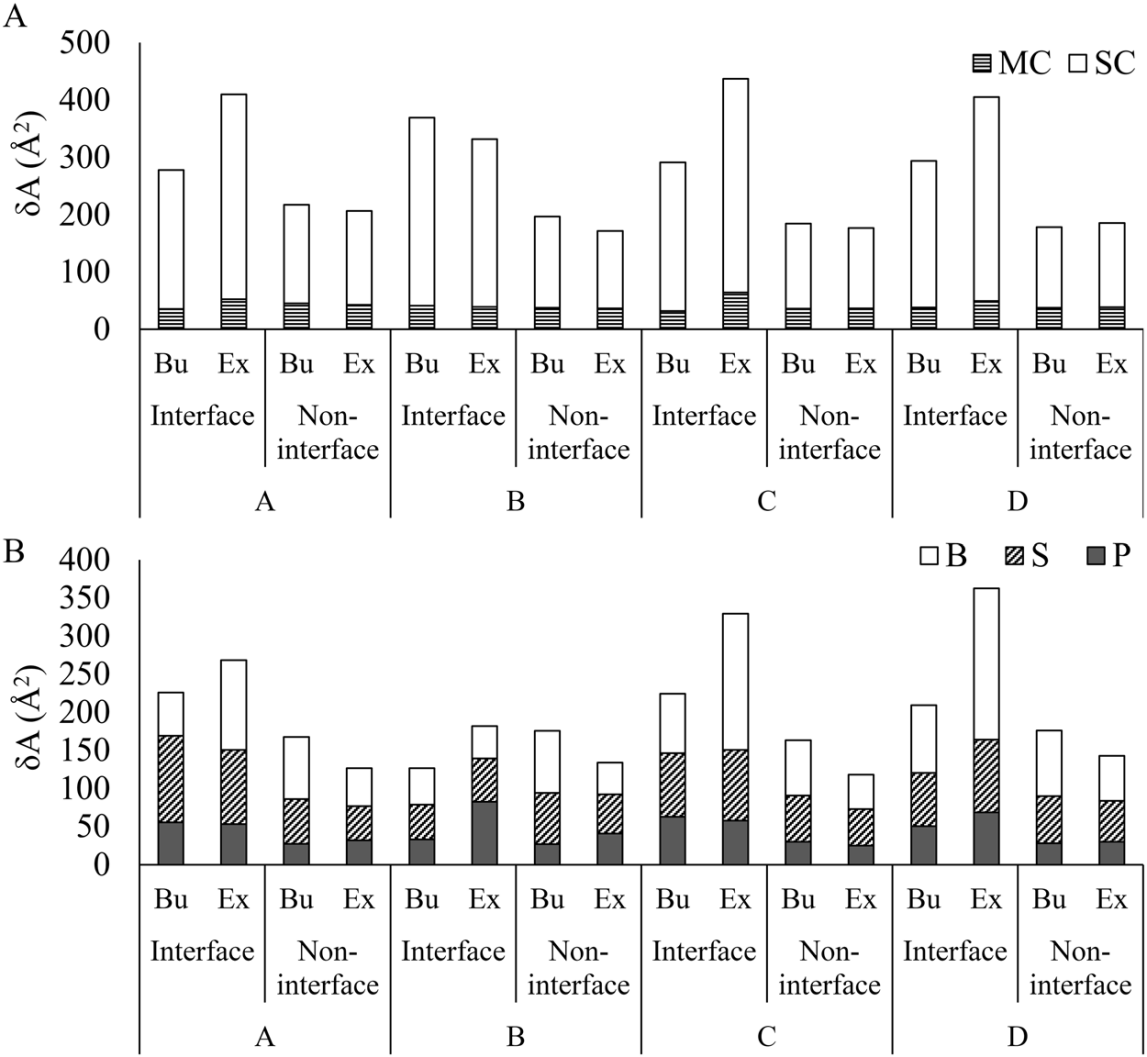
Distribution of δA in main chain and side chain calculated on 116 RBPs **(A)**, and in phosphate, sugar and bases calculated on 31 RNAs **(B)**. The average values are presented for Buried (Bu) and Exposed (Ex) surfaces in interface and in non-interface regions of different class of complexes.

The distributions of δA_P_ and δA_R_ at the interface and at the non-interface regions are shown in Fig. 1B and 1C, respectively. At the non-interface region, the majority (86%) of $\delta {A}_{P}^{non \mbox{-} \mathrm{int}}$ remain within the range between −50 Å^2^ and 50 Å^2^ (Fig. 1C). We find, irrespective of different classes, the change in accessibility at the interface region is always higher than the non-interface region (Fig. 2A). Buried (Bu: gain of accessibility) and exposed (Ex: loss of accessibility) surfaces of RBPs contribute almost equally to δA_P_ in the non-interface region. However, at the interface, exposed surface contribute 60% to $\delta {A}_{P}^{\mathrm{int}}$, whereas, the buried surface contributes only 40%. Therefore, the effective change remains higher at the interface compared to the non-interface region. About 80% of changes in δA_P_ both at the interface and the non-interface regions are contributed by the side chain atoms (Fig. 2A). The change in $\delta {A}_{P}^{\mathrm{int}}$ at the interface is highest in class D followed by class B, class A and class C. Except in class B, significant difference is observed between the exposed and the buried surfaces in all other class of interfaces. The δA_R_ values are always higher at the interface compared to the non-interface regions in the entire dataset as well as in different classes (Fig. 2B). At the interface, δA_R_ is the highest for the bases followed by the sugar and the phosphate.

### Effect of conformational change on accessibility

**Figure 3:**
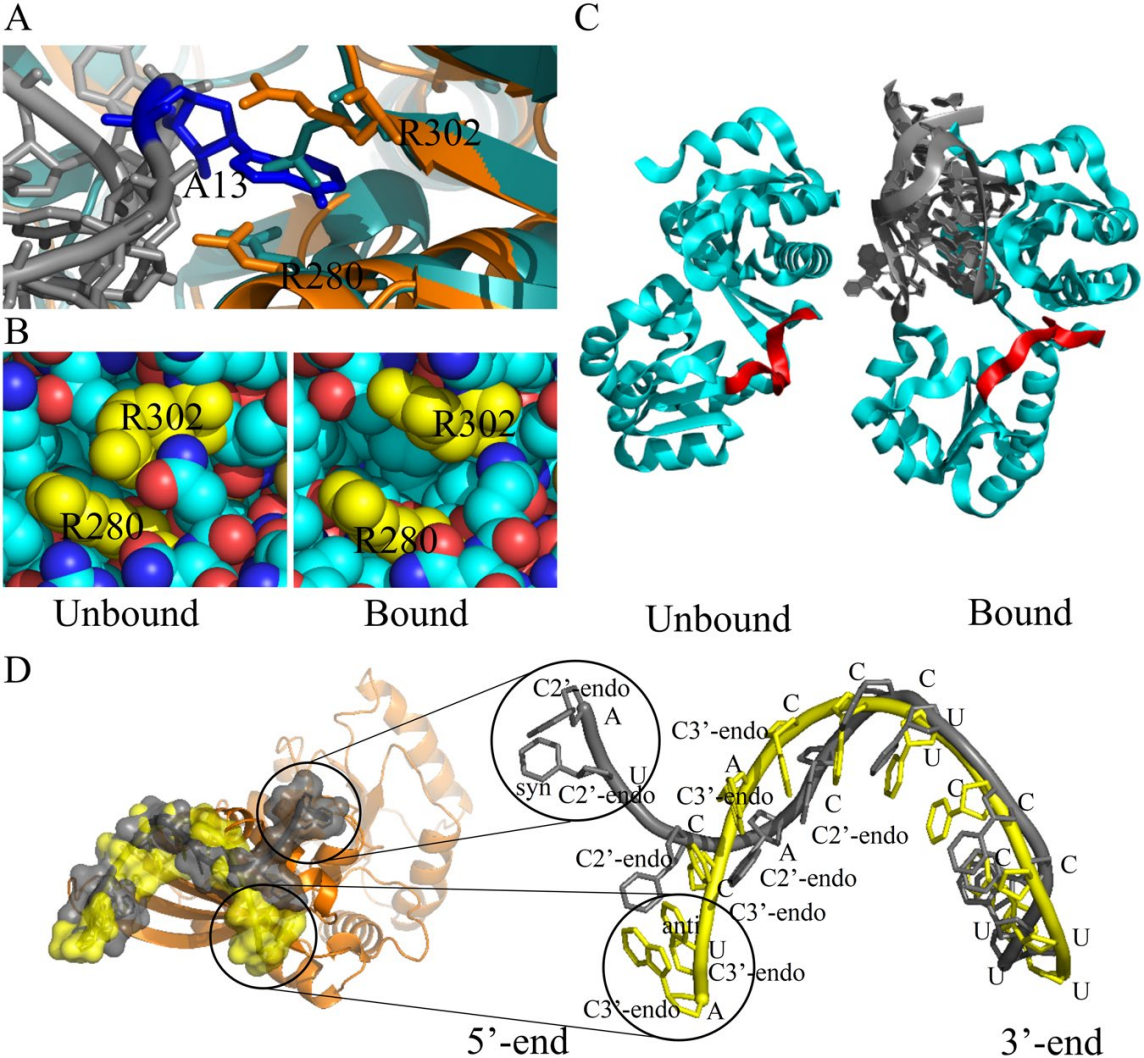
Change in accessibility on local and global conformational change. **(A)** Superposed structures of RNA splicing endonuclease in bound^42^ (in orange, PDB id: 2GJW) and in unbound^43^ (in cyan, PDB id: 1R0V) conformations with the RNA (shown in grey). Arg-nucleotide-Arg sandwich at the cleavage site of the nuclease is shown. Both the Arg are labeled and shown in stick. Change in conformation of R302 allows A13 (in blue) to protrude into the endonuclease pocket and stacked by the two Arg. **(B)** Top view of the aforementioned structure in sphere representation. Both the Arg are shown in yellow. In unbound structure, the endonuclease pocket is not accessible to the nucleotide. Change in conformation of R302 makes the pocket more accessible. **(C)** Unbound^44^ (PDB id: 1AD2) and bound^45^ (PDB id: 2HW8) structures of ribosomal protein L1 (in cyan). The loop at the hinge region connecting two domains is colored in red. RNA molecule in the bound structure is shown in grey cartoon. **(D)** Superposed structures of unbound (PDB id: 1SDR, in yellow) and bound (PDB id: 3IEV, in grey) forms of 12-nucleotides long 3′-end of 16 S rRNA with ERA. Protein is represented in orange cartoon.

Conformational changes between unbound and bound forms are estimated in terms of *i*-rmsd, which is the root mean squared deviation of interface C_α_ and P atoms of amino acids and nucleotides, respectively. Based on the degree of conformational changes, the protein-RNA binding can be classified into rigid body (*i*-rmsd < 1.5 Å), semi-flexible (*i* -rmsd within 1.5 Å to 3.0 Å) and full flexible (*i*-rmsd > 3.0 Å)^11,13^. Although we find the average change in $\delta {A}_{P}^{\mathrm{int}}$ is −96 Å^2^ and −100.4 Å^2^ for rigid-body and semi-flexible bindings, respectively, the change is significantly higher (−248 Å^2^) for full flexible binding. We find a moderate correlation (R = 0.6) between $\delta {A}_{P}^{\mathrm{int}}$ and *i*-rmsd. Besides, we also find the change in interface accessibility is significantly contributed by the side chain conformations (Fig. 2A), which is ignored in *i*-rmsd calculation. This can be exemplified in Fig. 3A,B, where the tRNA splicing endonuclease undergoes rigid body association (*i*-rmsd is 1.0 Å), however, its interface shows a significant change in accessibility ($\delta {A}_{P}^{\mathrm{int}}$ is −410.7 Å^2^) upon binding with its partner RNA. Here, the side chain ($\delta {A}_{P}^{\mathrm{int}}$ is −356 Å^2^) accounts for the large change in accessibility than its main chain ($\delta {A}_{P}^{\mathrm{int}}$ is −54.6 Å^2^). Counter examples are also observed, where the small change in interface accessibility does not correlate with the high *i*-rmsd values. This is exemplified in ribosomal L1 protein, which undergoes significant conformational change (*i*-rmsd is 5.1 Å) upon binding with its partner RNA even though the change in accessibility is only −2.2 Å^2^. The N- and C-terminal domains of L1 are linked by a short and a long loop (Fig. 3C). In the unbound form, the buried surface area between these two domains is very small. Upon binding with RNA, the long loop acts as a hinge and moves both the domains apart to facilitates the RNA binding. This domain movement leads to higher *i*-rmsd without affecting the overall change in accessibility. Similarly, changes in accessibility may also be attributed to the backbone as well as to the conformational changes of sugar and bases of RNA. For an instance, *E*. *coli* Ras-like protein (ERA), which acts as a chaperone for folding and maturation of 16S rRNA induces a large conformational change in 12-nucleotides long 3′-end of 16 S rRNA. The RNA adopts a Z-like structure upon binding with the KH domain of ERA^14^, and the estimated $\delta {A}_{R}^{\mathrm{int}}$ is −311.7 Å^2^. The second U from the 5′-end of the 12-nucleotides sequence changes the conformation of the base (anti-to-syn) and the sugar pucker (C2′-endo-to-C3′-endo), and contributes −96.5 Å^2^ change in accessibility (Fig. 3D).

### Changes in secondary structural elements in RBPs upon binding

**Figure 4:**
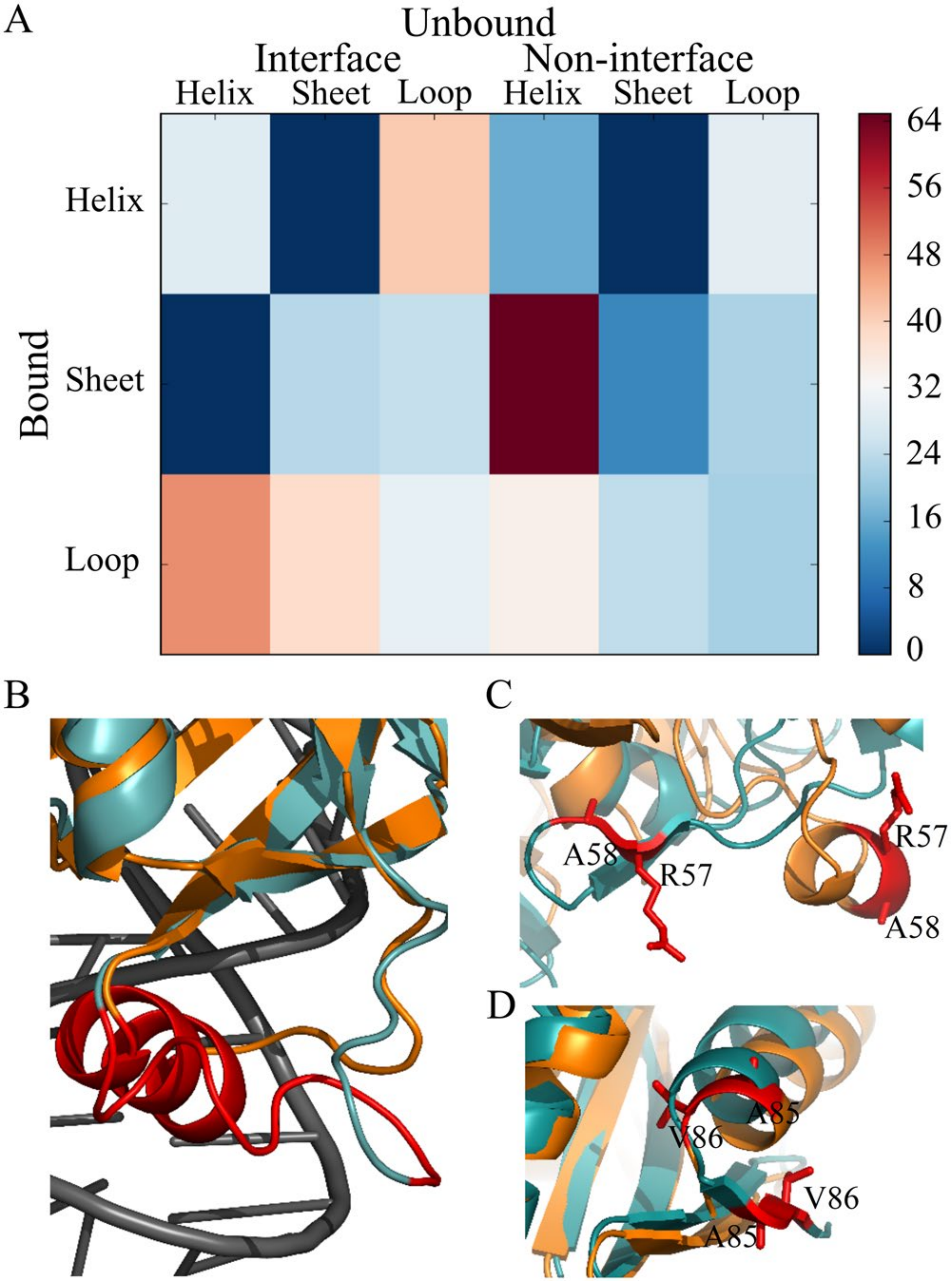
Changes in |ΔA| due to the transitions of secondary structural elements in RBPs upon binding with RNA. **(A)** Average |ΔA| calculated per transition is presented for both interface and non-interface regions. **(B)** A loop-to-helix transition. Here, the α1-helix of L25 (Lys14 to Ala23, coloured in red) is unstructured in the unbound state (PDB id: 1B75), which adopts a helical conformation upon binding with the major groove of 5 s rRNA (PDB id: 1DFU). **(C)** A sheet-to-helix transition. Here, Arg57 and Ala58 (shown in red stick) of translation elongation factor EF-Tu are in sheet conformation in the unbound state (PDB id: 1TUI), which adopt helical conformations upon binding with the tRNA (Cys) (PDB id: 1B23). **(D)** Another example of sheet-to-helix transition. Here, Ala85 and Val86 (shown in red stick) of CCA-adding enzyme are in β-sheet conformations in the unbound state (PDB id: 1UET) of the enzyme, which adopt α-helical conformations upon binding with the t-RNA (PDB id: 2DRB). In all these figures, the protein in bound and unbound states is shown in orange and teal, respectively, and the RNA is shown in grey.

Conformation changes can alter the secondary structures during unbound to bound transition leading to the change in accessibility. Figure 4A shows the average |ΔA_P_| accounts for different types of transitions in the secondary structural elements upon binding. We find the average change in accessibility at the interface is highest (|ΔA_P_| = 47.5 Å^2^) in transitions from loop-to-helix followed by in transitions from helix-to-loop (|ΔA_P_| = 41 Å^2^) and from loop-to-sheet (|ΔA_P_| = 38.4 Å^2^). Figure 4B shows an example of loop-to-helix transition where the unstructured α1-helix of L25 protein in the unbound state (PDB id: 1B75) adopts the helical conformation upon binding with the major groove of 5 s rRNA (PDB id: 1DFU)^15^. The α1-helix loses 230 Å^2^ upon binding with its partner RNA. We did not find any transition from helix-to-sheet or vice-versa at the interface.

At the non-interface region, the highest change in accessibility is observed in transitions from sheet-to-helix (|ΔA_P_| = 64.9 Å^2^). This change is observed in the following four residues from two different RBPs. Two residues, Arg57 and Ala58 in translation elongation factor EF-Tu (PDB id: 1TUI), undergo sheet-to-helix transitions upon binding with the tRNA(Cys) (PDB id: 1B23) (Fig. 4C). The other two residues, Ala85 and Val86 in the unbound state of the CCA-adding enzyme (PDB id: 1UET), undergo sheet-to-helix transitions upon binding with the tRNA (PDB id: 2DRB) (Fig. 4D). Loop-to-helix transitions also contributes significantly to the change in accessibility (average |ΔA_P_| = 34.3 Å^2^) at the non-interface regions, whereas, transitions from helix-to-loop or loop-to-sheet contribute moderately.

### The effect of intermolecular H-bonds on accessibility

**Figure 5:**
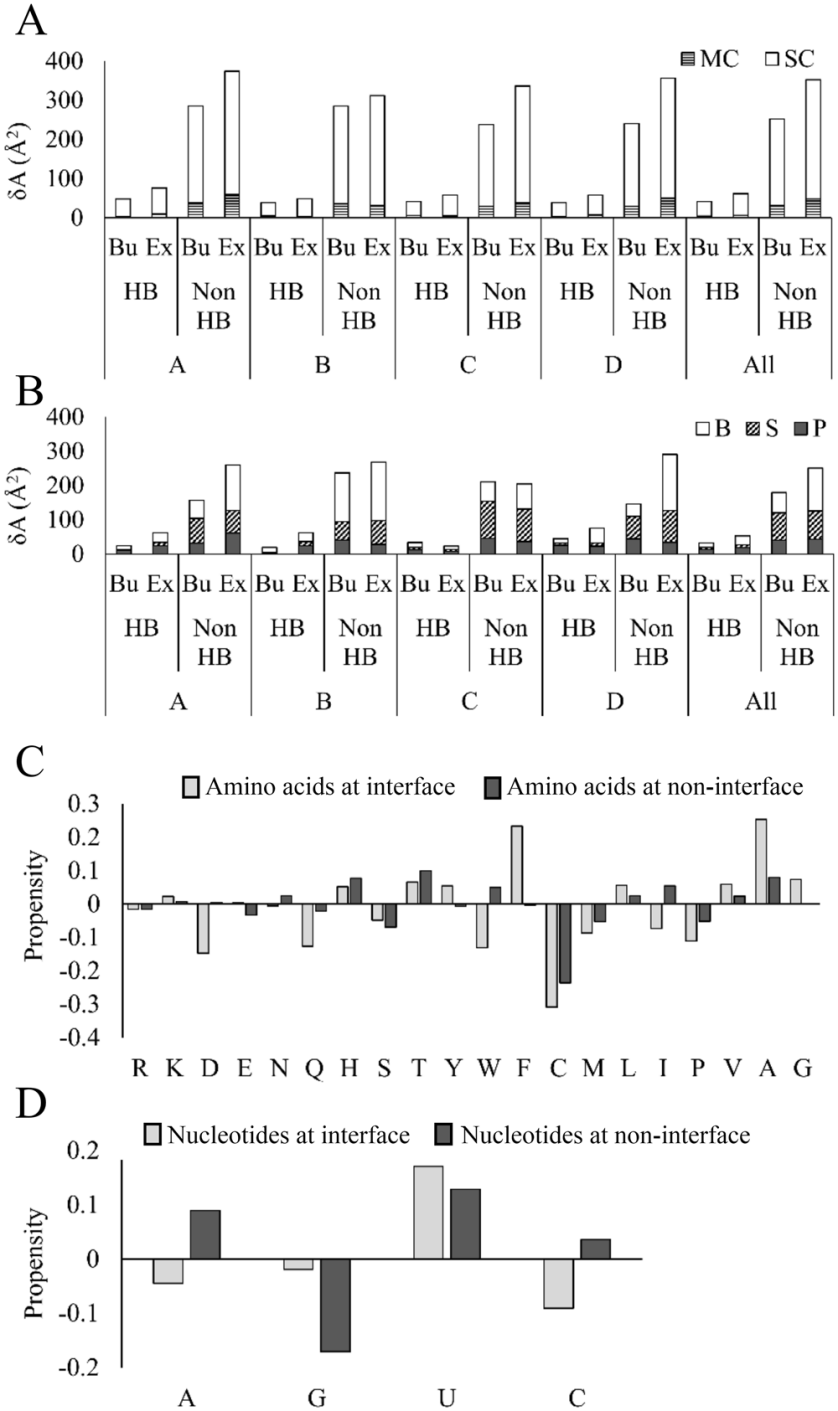
Distribution of δA in main chain and side chain calculated on 116 RBPs **(A)**, and in phosphate, sugar and bases calculated on 31 RNAs **(B)**. The average values are presented for Buried (Bu) and Exposed (Ex) surfaces of different class of complexes. Values for both H-bond (HB) and non-H-bond (Non HB) residues are given. Propensities of **(C)** amino acid residues and **(D)** nucleotides to get exposed or buried upon binding.

We evaluate the effect of intermolecular H-bonds on the change in solvent accessibility of amino acid residues and nucleotides at the protein-RNA interfaces. We find the change in accessibility is significant for the residues that are not involved in any H-bond with the partner nucleotides across the interfaces as compared to those involved in H-bond (Fig. 5A). This trend is observed in the entire dataset as well as among the different classes. The average |δA_P_| is 61.3 Å^2^ for residues involved in H-bonds across the interface, whereas, those do not participate in H-bonds have an average of 93 Å^2^.

At the RNA side, the change in accessibility is significantly higher for nucleotides that do not involved in any H-bond compared to those involved in H-bond across the interface (Fig. 5B). This phenomenon is observed in the entire dataset as well as among the different classes. Interestingly, different trend is observed in |δA_R_| among phosphate, sugar and bases. Among those involved in H-bonds across the interface, the highest change in average |δA_R_| is observed in bases (38.3 Å^2^), followed by phosphate (32.5 Å^2^) and sugar (14.4 Å^2^). On the contrary, those do not participate in any H-bonds across the interface, the highest change in average |δA_R_| is observed in bases (183.7 Å^2^), followed by sugar (163 Å^2^) and phosphate (83.5 Å^2^).

### Accessibility of residues and nucleotides upon binding

The propensity of amino acid residues to get buried or exposed upon binding is shown in Fig. 5C. Upon binding, a positive propensity signifies that the residue prefers to get exposed while a negative propensity indicates their preference to get buried. Among the positively charged residues, Arg shows little preference to get buried both at the interface and at the non-interface regions, while, Lys shows opposite trend at both the regions. Among the negatively charged residues, Asp shows strong preference to get buried at the interface, while, Glu shows similar preference at the non-interface region, but with a lesser extent. Between Asn and Gln, the former shows preference to get exposed only at the non-interface, while the later shows preference to get buried both at the interface and at the non-interface regions. Among the neutral polar residues, His and Thr prefer to get exposed, whereas, Ser prefers to get buried both at the interface and at the non-interface regions. Among the three aromatic residues, Tyr and Phe both prefer to get exposed at the interface with a different magnitude, while Trp prefers to get buried at the interface and get exposed at the non-interface. Both the sulphur containing residues, Cys and Met, prefer to get buried both at the interface and the non-interface regions, however, with a different magnitude; the former have stronger preference than the later. Among the hydrophobic residues, Leu, Val and Ala prefer to get exposed both at the interface and the non-interface regions, while Gly prefers to get exposed only at the interface. On the contrary, Pro prefers to get buried both at the interface and the non-interface regions. Ile behave differently, it prefers to get buried at the interface and get exposed at the non-interface regions.

Among the four nucleotides, adenine and cytosine prefer to get buried at the interface and get exposed at the non-interface regions. Guanine prefers to get buried, while uracil prefers to get exposed both at the interface and at the non-interface regions (Fig. 5D).

### Change in SASA can be used as a parameter to score protein-RNA decoys

**Figure 6:**
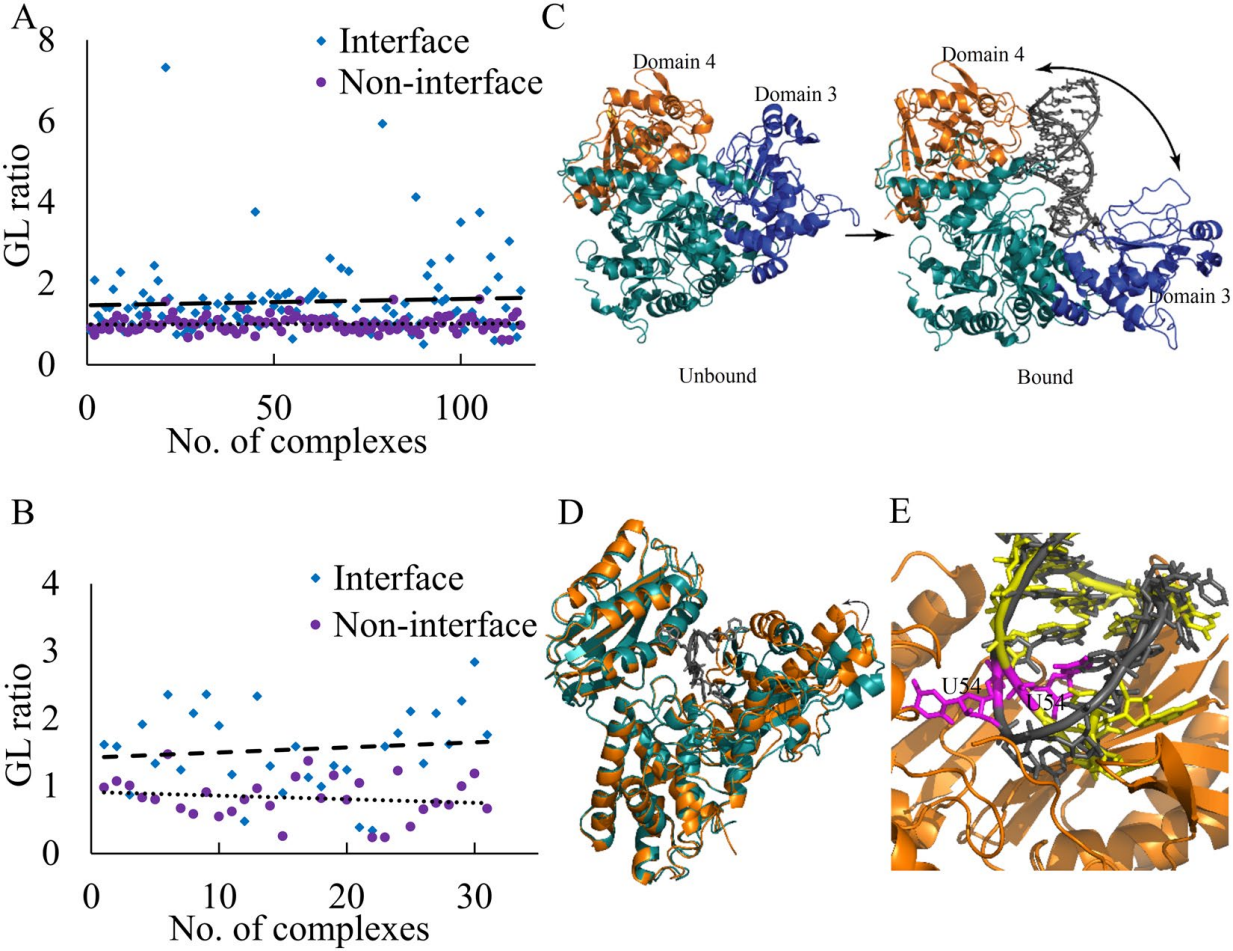
Gain or loss in accessibility. **(A)** The distribution of GL ratio of RBPs at interface and non-interface regions. **(B)** The distribution of GL ratio of RNAs at interface and non-interface regions. **(C)** In the unbound state of IRP1(PDB id: 2B3Y), domain 3 and 4 are in closed conformation, which transformed into open conformation upon binding with the RNA (PDB id: 3SNP). Both the domains move apart (bidirectional arrow), thereby increasing significant amount of surface to accommodate the RNA. Domain 3 and 4 are colored in blue and orange respectively, and the rest of the protein is colored in teal. **(D)** Example of “open-to-close” conformation change in poly(A) polymerase and oligo(A) RNA complex (PDB id: 2Q66). In the unbound state (color teal; PDB id: 2HHP), the binding cleft between N- and C-terminal domains remain wide open, which transformed into closed conformation upon binding with the RNA, hence losing the accessibility. **(E)** Superposed T-arm analogue RNA segment in bound (in grey; PDB id: 3BT7) and in unbound (in yellow; PDB id: 1EVV) states. The U54 (in magenta) in the unbound state remains inside the loop, which flips out to the active site upon binding with the 5-methyluridine methyltransferase TrmA (shown in orange).

Binding induced conformational transitions lead to change in SASA of individual atoms in interacting subunits. Few of the atoms gain accessible surface and few lose. We find the average gain to loss ratio of accessible surface area (GL ratio) upon binding is 1.7 and 1.0 (p-value = 1.6E-04, single tailed t-test) at the interface and at the non-interface regions, respectively. In majority of the cases, the ratio is close to one at the non-interface region. This ratio has never been used in any available protein-RNA docking algorithms^16^, and may be efficiently use to score the flexible docking models to identify the near native solution. Figure 6A and 6B shows the distribution of the GL ratio in 115 RBPs and in 31 RNAs, respectively. The highest GL ratio (18.7) is found in the structure of iron regulatory protein 1 (IRP1) in complex with ferritin H IRE RNA (PDB id: 3SNP). This high ratio can be attributed to the large conformational change in IRP1 upon binding with the RNA, which is facilitated by a major rearrangement of the two domains of IRP1^17^ (Fig. 6C), gaining 1279 Å^2^ accessibility at the interface. The lowest GL ratio (0.5) is observed in complex between poly(A) polymerase and oligo(A) RNA (PDB id: 2Q66). In the polymerase, the catalytic site is located at the bottom of the cleft between N- and C-terminal domains of the polymerase^18^. In the unbound state, both the domains of the polymerase remain in open conformation and adopt closed conformation upon binding with the RNA, thereby losing 163.6 Å^2^ surface area at the interface (Fig. 6D). The highest GL ratio (2.8) at the RNA binding surface is observed in the T-arm analogue RNA segment (PDB id: 1EVV) in complex with 5-methyluridine methyltransferase TrmA (PDB id: 3BT7). In the unbound state, U54 remains buried inside the T-loop of the tRNA and forms a reverse-Hoogsteen base pair with A58^19^. In the bound state, the loop changes its conformation and U54 flips out towards the active site of the enzyme, thereby gaining the surface accessibility of 310.4 Å^2^ (Fig. 6E).

## Discussion

Proteins and RNAs are flexible biomolecules that often undergo conformational changes upon binding. These conformational changes affect their solvent accessibility at the bound state as compared to their unbound states. Moreover, solvent accessibility can be directly attributed to the overall entropy of the system^5,6^, and any change in accessibility upon complexation may contribute to the free energy of binding. Hence, it is utmost important to understand how the accessibility changes upon protein-RNA recognition. In this study, we have used the protein-RNA docking benchmark version 2^13^ to evaluate the change in accessibility at the binding interfaces as well as at the non-interface regions of the partner molecules. The 3D structures used in this study are solved either by X-ray crystallography or by NMR, and are determined by different groups. Hence, it may be subject to serious artefacts. However, Fig. 2B shows that in most of the complexes, the non-interface regions of the proteins display only small changes in accessibility. The changes occur at the non-interface regions in both directions and their differences in unbound and bound is insignificant. Thus, the increased accessibility observed at the interface in the complexes is probably not influenced by the experimental artefacts. In this study, all the calculations were performed on the equivalent atoms of the bound and the corresponding unbound structures. About 2% of all the amino acid residues were discarded from the bound structures due to the mismatches in the alignment with their unbound form. This affects the average B (reported in Table 1), which is smaller compared to the values calculated on the benchmark dataset^13^. Mismatches in the alignment may arise due to the missing residues, which can be attributed to the disorder to order transitions of the partner molecules upon binding^1,20^. This is exemplified in Figure 3B, which illustrates the superposed structures of L25 protein in unbound (PDB id: 1B75) and in bound (PDB id: 1DFU) conformations with 5S rRNA. Residues 14 to 23 in L25 interact with the major groove of the RNA. This stretch of residues is unstructured in the unbound state, and changes to an α-helix in the bound state^15^. We have identified 10 interface residues in L25 that undergo loop-to-helix transitions upon binding with the rRNA with ΔA_P_ of 230 Å^2^. Another example of disorder to ordered transition is found in the nuclear RNA exporter protein (TAP-NTD; PDB id: 3RW6) bound with the constitutive transport element (CTE) RNA (PDB id: 3RW7)^21^. Here, the linker of TAP-NTD, spanning from Ser194 to Leu204 (crystallographic data is missing from residue 199 to 202), is disordered in the unbound state. When bound to CET, the linker is well ordered and reorients the RRM and LRR domains in favorable binding conformation. This disorder to order transition accounts for a significant change in overall accessibility at the interface ($\delta {A}_{P}^{\mathrm{int}}$ = −170 Å^2^).

Protein–RNA binding may results in four different possible changes in the solvent accessibility: the binding surfaces of both RBPs and RNAs get exposed or buried, RBPs get exposed but RNAs get buried, and vice-versa. Among the 21 P_U_R_U_ cases, we find in nine cases both the interacting surfaces get exposed upon binding, in five cases protein interfaces get exposed and RNA interfaces get buried, while in rest of the cases, RNA interfaces get exposed and protein interfaces get buried. This phenomenon can be correlated with the concept of induced fit in protein–RNA recognition^1^. We did not find any example where both protein and RNA interfaces get buried simultaneously upon binding. Negative δA indicates a gain in accessibility upon binding, which can be attributed to the exposure of molecular surfaces. On the other hand, a positive δA indicates loss in accessibility upon binding, which can be attributed to the burial of the molecular surfaces. The average δA_P_ (−120.5 Å^2^, Table 1) suggests overall gain of solvent accessibility at the interface region of RBPs upon binding with RNAs, a phenomenon which is also observed in protein-protein recognition^8,22^. Except in two cases, the average δA_P_ and δA_R_ (Table 1) indicates that both RBPs and RNAs gain accessibility at the protein–RNA interfaces upon binding. Among two exceptions, in one case, the positive value of average δA_P_ in class B complexes can be attributed to the unstructured regions (Fig. 3B) along with the elongated N- and C-terminal loops in the majority of the ribosomal proteins, which are stabilized while forming the ribosomal assemblies^23^. In another case, the positive value of average δA_R_ at the interfaces in class C complexes can be supported by the molecular dynamics simulation study of double-stranded RNA, which explains that a stable A-form geometry of duplex RNA undergoes negligible changes while interacting with double-stranded RNA binding domains^24^.

The interface regions undergo significant change in accessibility compared to the non-interface regions both in RBP and RNA. In RBPs, side chain atoms always contribute more to δA compared to main chain atoms. This can be attributed to the higher degrees of freedom of side chain atoms than the main chain. A moderate correlation (R = 0.6) between $\delta {A}_{P}^{\mathrm{int}}$ and *i*-rmsd^11^ signifies that the side chain also play an important role in RNA recognition. The large movements of interacting domains in RBPs upon binding may or may not justify the large change in accessibility. Domains with large inter-domain buried area generally contribute to high $\delta {A}_{P}^{\mathrm{int}}$ that is correlated with high *i*-rmsd. On the other hand, the degree of change in accessibility does not always correlate with the *i*-rmsd if the interacting domains are connected with unstructured loop and the inter-domain buried surface area is small. In such cases, side chain conformations play the key role in change in accessibility. The change in accessibility can also be described in terms of binding free energy of the RBPs. Experimentally determined Gibbs free energy (Δ*G*), curated by Barik *et al*.^10^, does not show any correlation with $\delta {A}_{P}^{\mathrm{int}}$(we ignored the positive or negative sign of $\delta {A}_{P}^{\mathrm{int}}$ for this calculation). However, a moderate correlation (R = 0.5) is observed in 17 cases where the *i*-rmsd is greater than 1.0 Å. Moreover, the correlation slightly improves (R = 0.53) for eight cases where the *i*-rmsd is greater than 2.0 Å. This can be attributed to the collaborative effects of hydrophobic collapse and hydrophilic exposures of the interacting atoms that contribute in binding free energy. This observation can be supported with a study by Janin^25^, which suggests that binding affinity of protein-protein interactions can be estimated by using only two geometric quantities: size of the interface and *i*-rmsd between bound and unbound forms of the interacting subunits. However, there are more accurate physics based empirical approaches to calculate Δ*G*, but the complexity of those algorithms make it computationally expensive and time consuming. At the interface, the extent of exposed surface of RBPs upon binding is always significantly higher than the buried surface. On the other hand, no such significant difference is observed at the non-interface region. Similar trend is observed at the binding surface of the RNA; in 81% of cases, RNA gain accessibility upon binding. On the other hand, in majority of the cases (71%), non-interface region of the RNA lose accessibility upon binding.

The extent of gain and loss of solvent accessibility upon binding is almost equal at the non-interface region. On contrary, a significant change (either gain or loss) in solvent accessibility is observed at the interface region. This can be attributed to the exclusion of water molecules from the binding site^26^. Consequently, the hydrophobic residues get exposed and subsequent contacts between RBPs and RNAs make a stable conformation. Moreover, the predominant presence of positively charged and aromatic amino acid residues^27^, having comparatively large and bulky side chains, provide higher degrees of flexibility at the interface compared to the non-interface region. Recently, many theoretical models have been developed to predict binding affinities and to discriminate the near native structures from the protein-protein decoys using MM-PBSA and MM-GBSA methods^28–30^. In both the approaches, the non-polar term directly correlates to the solvent accessible surface area or SASA. HawkRank, a scoring function developed by Feng *et al*.^31^ describes a method to implement SASA-based solvation model to identify the near-native complexes from protein-protein decoys. In a similar context, GL-ratio can be used as a parameter to score the protein-RNA decoys generated by flexible docking algorithms. However, more detailed studies are required to optimize this parameter.

We observe frequent transitions of secondary structural elements in RBPs upon binding with its partner RNA. In the entire dataset, we find 308 transitions at the interface and 2,088 transitions at the non-interface regions. At the interface, loop-to-helix transitions are the most abundant followed by helix-to-loop, loop-to-sheet and sheet-to-loop. Loop-to-helix transition is frequently observed in RBPs, and RNA plays an important role to induce the folding^32^. Except the transitions from loop-to-sheet, all other transitions account for a negative change in ΔA_P_, which signifies a relative gain in accessibility of interface residues upon binding (Supplementary Table S2). Interestingly, all the residues that retain their secondary structures at the interface gain accessibility upon binding. At the non-interface region, all the transitions, except helix-to-loop, account for a loss in accessibility. The average ΔA_P_ per residue is highest for sheet-to-helix transitions, which accounts for only 0.2% of all the transitions, followed by sheet-to-loop and loop-to-sheet transitions. Average ΔA_P_ for loop-to-helix transition is significantly low though it accounts for 32% of all the transitions at the non-interface region. The secondary structures of the non-interface regions, which remain unchanged upon binding, have significantly lower average ΔA_P_ compared to the interface regions (Supplementary Table S2). In the entire dataset, we do not observe any helix-to-sheet transitions both at interface and non-interface, and sheet-to-helix transitions at interface region.

Among the specific interactions, electrostatic forces between the positively charged amino acids and the negatively charged phosphate backbone of nucleotides have long range attractions, which “lure” the RBPs and their partner RNAs^27,33^. On the other hand, H-bonds are effective in short range contacts and play a crucial role in specific protein-RNA recognition^34^. In this study, we observe the interacting residues and nucleotides that are not involved in intermolecular H-bonds show significant change in accessibility upon binding compared to those involved in such interactions. Moreover, the positively charged amino acids, which are prevalent at the protein–RNA interfaces, display low propensity of gaining or losing accessibility. Uracil and guanine prefer to gain and lose accessibility, respectively both at the interface and the non-interface regions. This may be attributed to their preferential binding with the RBPs^27^.

## Materials and Methods

### Dataset of protein-RNA complexes and their unbound structures

Protein–RNA complexes and their unbound structures were taken from the protein–RNA docking benchmark version 2^13^. Modified residues and nucleotide bases in each structure were kept with their corresponding amino acids and bases by changing the keyword ‘HETATM’ to ‘ATOM’ in their atomic coordinate files taken from the Protein Data Bank (PDB)^35^. Each of the PDB file was cleaned following Barik *et al*.^36^. According to Bahadur *et al*.^34^, the dataset was divided into four classes: (A) complexes with tRNA, (B) complexes with ribosomal proteins, (C) complexes with duplex RNA and (D) complexes with single-stranded RNA.

### Calculation of solvent accessibility

The size of the protein–RNA interface (B) was estimated by measuring SASA buried in contact. We calculated B using the following two equations:

1 $${B}^{B}={A}_{P}^{B}+{A}_{R}^{B}-{A}_{PR}^{C}$$

2 $${B}^{U}={A}_{P}^{U}+{A}_{R}^{U}-{A}_{PR}^{C}$$

where A_P,_ A_R_ and A_PR_ are the SASA of protein, RNA and protein-RNA complex, respectively. In the first equation, SASA values were calculated using the interacting partners taken from the complex, while in the second equation, they were calculated using their unbound structures. The difference in B upon binding is further calculated using the following equation:

3 $${\rm{\Delta }}B={B}^{U}-{B}^{B}={\rm{\Delta }}{A}_{P}+{\rm{\Delta }}{A}_{R}$$

where the corresponding change in accessibility of protein and RNA upon binding can be given by the following two equations:

4 $${\rm{\Delta }}{A}_{P}={A}_{P}^{U}-{A}_{P}^{B}$$

5 $${\rm{\Delta }}{A}_{R}={A}_{R}^{U}-{A}_{R}^{B}$$

Equations 4 and 5 quantify the effect of any conformational change on SASA upon binding in protein and RNA structures, respectively. We used the following equations to normalize the above values:

6 $$\delta {A}_{P/R}^{\mathrm{int}}=\frac{\Delta {{A}^{\mathrm{int}}}_{P/R}}{{{B}^{B}}_{P/R}}\times 1000\,{{\rm{\AA }}}^{{\rm{2}}}$$

7 $$\delta {A}_{P/R}^{non \mbox{-} \mathrm{int}}=\frac{\Delta {{A}^{non \mbox{-} \mathrm{int}}}_{P/R}}{{{A}^{B}}_{P/R}}\times 1000\,{{\rm{\AA }}}^{{\rm{2}}}$$

where $\delta {A}_{P/R}^{\mathrm{int}}$ represent the normalized value at the protein or the RNA interface (${\rm{\Delta }}{A}_{P/R}^{\mathrm{int}}$) and $\delta {A}_{P/R}^{non \mbox{-} \mathrm{int}}$ represent the same at the non-interface region $({\rm{\Delta }}{A}_{P/R}^{non \mbox{-} \mathrm{int}})$. SASA values were calculated using the program NACCESS^37^, which implements the Lee and Richards^4^ algorithm. Interface area was calculated using PRince^38^. Any atom belongs to amino acid residues or nucleotides is considered at the interface if SASA is lost upon binding. Non-interface regions are protein or RNA surfaces that are not included in the interface. All these calculations were performed over the equivalent atoms of the bound and their corresponding unbound structures of the interacting partners. The average values of SASA of interface (*B*^*B*^) and non-interface (*A*^*B*^) regions for each class of complexes are mentioned in Table 1. Pairwise alignment between the bound and the unbound structures of the interacting partners were carried out using ClustalW^39^. Secondary structures were assigned using the program DSSP^40^. According to Rost and Sander^41^, α-helix, 3_10_-helix and π-helix were categorized as helices (H), extended strand as strands (S) and isolated β-bridge, turn, bend and coils as loops (L). However, an isolated β-bridge (B) proceed by a coil (_) together considered as two strands (B_ = SS), whereas, two isolated β-bridge with a coil in between is considered as three loops (B_B = LLL).

The propensity of an amino acid residue or nucleotide to get exposed or buried upon binding was calculated using the following equation:

8 $${P}_{i}=\,\mathrm{ln}\,\frac{{f}_{i}{\rm{\Delta }}{A}^{\exp }}{{f}_{i}{\rm{\Delta }}{A}^{bur}}$$

where

$${f}_{i}{\rm{\Delta }}{A}^{\exp }=\frac{{\rm{\Delta }}{A}_{i}^{\exp }}{\underset{i=1}{\overset{n}{{\rm{\Sigma }}}}{\rm{\Delta }}{A}^{\exp }}\,{\rm{and}}\,{f}_{i}{\rm{\Delta }}{A}^{bur}=\frac{{\rm{\Delta }}{A}_{i}^{bur}}{\underset{i=1}{\overset{n}{{\rm{\Sigma }}}}{\rm{\Delta }}{A}^{bur}}$$

here, Δ*A*_*i*_^*exp*^ and Δ*A*_*i*_^*bur*^ are gain or loss in accessibility of amino acid or nucleotide of type *i* upon binding, respectively.

### Data Availability

The data that support the findings of this study are available from the corresponding author RPB on request.

## Conclusion

We evaluate the change in SASA of RBPs and their partner RNAs upon binding. We find in majority of the cases, the interface of both the binding partners gain accessibility upon binding. On the other hand, majority of RNA non-interface region lose accessibility. Interestingly, no such significant bias is observed at the non-interface region of RBPs. The change in accessibility is grossly attributed to the side chain conformation even though a moderate role of main chain conformation is also observed. Additionally, the significant change in accessibility is observed when the binding is more flexible including large domain movements that expose the inter-domain buried surfaces in RBPs. Besides, we also observed a significant change in accessibility associated with secondary structural transitions at the interface of RBPs upon binding. Close-to-open transitions upon binding lead to gain in accessibility, whereas, accessibility is lost in open-to-close transitions. The significant change in accessibility at the binding interface is an intrinsic feature of both the binding partners governed by the binding induced flexibility in protein-RNA recognition, and may have implications in designing flexible docking algorithms.

## Electronic supplementary material

Supplementary Information
