# Supplementary material for: An account of solvent accessibility in protein-RNA recognition
Source: Sci Rep. 2018 Jul 12;8:10546. doi: 10.1038/s41598-018-28373-2 (PMC6043566; doi:10.1038/s41598-018-28373-2)
Supplement: Supplementary file 1 — Supplementary Information [file 41598_2018_28373_MOESM1_ESM.docx]

Supplementary Information

An account of solvent accessibility in protein-RNA recognition

Sunandan Mukherjee and Ranjit Prasad Bahadur*

Computational Structural Biology Laboratory

Department of Biotechnology, Indian Institute of Technology Kharagpur

Kharagpur-721302, India

*Corresponding author

E-mail: [r.bahadur@hijli.iitkgp.ernet.in](mailto:r.bahadur@hijli.iitkgp.ernet.in); [ranjitp_bahadur@yahoo.com](mailto:ranjitp_bahadur@yahoo.com)

Ph: +91-3222-283790

Fax: +91-3222-27870

## **Table S1:** Dataset of protein-RNA complexes and their unbound subunits

| **PDB ids^a^** | | | **Resolution** | **Class** | **B** | **ΔA_P_** | **ΔA_P_^int^** | **ΔA_P_^non-int^** | **δA_P_^int^** | **δA_P_^non-int^** | **ΔA_R_** | **ΔA_R_^int^** | **ΔA_R_^non-int^** | **δA_R_^int^** | **δA_R_^non-int^** |
| --- | --- | --- | --- | --- | --- | --- | --- | --- | --- | --- | --- | --- | --- | --- | --- |
| **Complex ID** | **Unbound protein** | **Unbound RNA** |  |  |  |  |  |  |  |  |  |  |  |  |  |
| **i) P_U_R_U_ (21 Cases)** | | | | | | | | | | | | | | | |
| 1ASY (A:R) | 1EOV:A | 2TRA:A | 2.9 | A | 3659.1 | 582.0 | 140.8 | 441.2 | 78.2 | 19.9 | -234.6 | -272.1 | 37.4 | -146.4 | 3.5 |
| 1B23 (P:R) | 1TUI:A | 1U0B:A | 2.6 | A | 2612.8 | 1006.1 | -394.6 | 1400.8 | -318.6 | 89.6 | -263.1 | -132.8 | -130.3 | -96.6 | -11.4 |
| 1C0A (A:B) | 1EQR:A | 1EFW:C | 2.4 | A | 3464.2 | 481.1 | -114.1 | 595.3 | -67.4 | 22.3 | 30.7 | 35.4 | -4.7 | 20.0 | -0.5 |
| 1DFU (P:MN) | 1B75:A | 364D:CB | 1.8 | B | 1688.2 | 70.6 | 118.4 | -47.8 | 142.9 | -9.4 | 134.0 | -71.7 | 205.7 | -83.4 | 32.7 |
| 1FEU (A:BC) | 2J01:Z | 364D:BC | 2.3 | B | 1589.7 | 1064.0 | 27.2 | 1036.8 | 34.1 | 107.3 | 160.8 | -38.3 | 199.1 | -48.4 | 31.1 |
| 1JBS (A:C) | 1AQZ:A | 1Q9A:A | 1.97 | D | 1235.6 | -191.0 | -214.8 | 23.8 | -346.1 | 3.2 | -530.0 | -175.8 | -354.2 | -285.8 | -77.4 |
| 1MMS (A:C) | 2K3F:A | 3I9C:A | 2.57 | B | 2429.2 | 57.0 | 67.1 | -10.2 | 56.5 | -1.5 | 384.6 | -42.6 | 427.2 | -34.3 | 61.3 |
| 1OOA (A:C) | 1LE5:B | 2JWV:A | 2.45 | C | 1909.6 | -561.4 | -160.9 | -400.5 | -161.5 | -25.3 | 55.8 | -253.3 | 309.1 | -277.4 | 73.5 |
| 1QTQ (A:B) | 1NYL:A | 3KNH:Y | 2.25 | A | 4541.1 | 524.1 | -55.8 | 579.9 | -26.0 | 27.0 | -455.2 | -634.5 | 179.3 | -265.2 | 17.2 |
| 1R3E (A:C) | 1ZE1:A | 1EHZ:A | 2.1 | C | 2909.8 | -966.3 | -190.3 | -776.0 | -141.3 | -53.9 | -233.5 | -301.4 | 67.9 | -192.8 | 35.0 |
| 1WSU (A:E) | 1LVA:A | 2RLU:A | 2.3 | D | 881.6 | 268.4 | 28.6 | 239.8 | 68.7 | 35.1 | 114.3 | -19.1 | 133.4 | -41.0 | 47.5 |
| 1ZBH (AD:E) | 1ZBU:AD | 1JU7:A | 3 | D | 738.2 | 266.6 | -142.0 | 408.6 | -359.9 | 19.1 | 177.6 | 50.5 | 127.1 | 146.9 | 43.3 |
| 2BH2 (A:C) | 1UWV:A | 1JZX:A | 2.15 | D | 4426.1 | 564.2 | 60.6 | 503.6 | 28.5 | 29.5 | -724.3 | -752.3 | 28.0 | -327.7 | 6.8 |
| 2BTE (A:B) | 1OBC:A | 2NQP:F | 2.9 | A | 636.0 | -34.5 | -27.1 | -7.4 | -91.5 | -0.2 | 568.3 | -28.6 | 596.9 | -84.3 | 74.9 |
| 2DRB (A:B) | 1UET:A | 3Q1Q:C | 2.8 | A | 2981.2 | 549.3 | -178.3 | 727.6 | -130.9 | 33.6 | 1136.3 | 35.8 | 1100.4 | 22.1 | 231.7 |
| 2FMT (A:C) | 1FMT:A | 3CW6:A | 2.8 | A | 2316.0 | -312.3 | -195.2 | -117.2 | -170.2 | -8.6 | -333.1 | -118.4 | -214.6 | -101.3 | -18.1 |
| 2R8S (LH:R) | 2HFF:AB | 1HR2:A | 2.81 | C | 1433.8 | 283.9 | -5.9 | 289.8 | -8.7 | 16.1 | -986.8 | -11.8 | -975.0 | -15.7 | -39.5 |
| 2ZZM (A:B) | 2YX1:A | 1WZ2:C | 2.65 | A | 3559.1 | -398.5 | -242.4 | -156.1 | -148.2 | -10.2 | 369.9 | 2.6 | 367.2 | 1.4 | 30.0 |
| 3HL2 (AA'BB':E) | 3BC8:AA'A''A''' | 3RG5:A | 2.81 | A | 1500.3 | -1875.9 | 0.9 | -1876.8 | 1.2 | -29.6 | -259.4 | -27.9 | -231.5 | -37.3 | -17.8 |
| 3SNP (A:C) | 2B3Y:A | 1AQO:A | 2.8 | C | 2234.6 | -2578.4 | -1279.2 | -1299.3 | -1217.3 | -39.0 | 11.2 | -80.2 | 91.4 | -67.8 | 27.9 |
| 3VJR (A:B) | 2PTH:A | INJM:5 | 2.4 | A | 553.1 | 87.2 | -76.3 | 163.4 | -270.6 | 18.3 | 61.1 | 86.3 | -25.2 | 318.1 | -5.8 |
| i**i) P_U_R_B_ (95 Cases)** | | | | | | | | | | | | | | | |
| 1AV6 (A:B) | 1B42:A |  | 2.8 | D | 841.0 | 63.3 | -37.7 | 101.1 | -97.6 | 7.7 |  |  |  |  |  |
| 1C9S (L:W1-7) | 2EXT:A |  | 1.9 | D | 1050.4 | 115.7 | 30.1 | 85.6 | 59.8 | 22.1 |  |  |  |  |  |
| 1CVJ (A:M) | 4F25:A |  | 2.6 | D | 1134.9 | -44.3 | -4.4 | -39.9 | -8.9 | -9.6 |  |  |  |  |  |
| 1F7U (A:B) | 1BS2:A |  | 2.2 | A | 5768.6 | -566.6 | 212.4 | -779.0 | 79.8 | -30.8 |  |  |  |  |  |
| 1G2E(A:B) | 4EGL:A |  | 2.3 | D | 2398.9 | 158.0 | -10.3 | 168.3 | -8.9 | 21.3 |  |  |  |  |  |
| 1H3E (AA':B) | 1H3F:AB |  | 2.9 | A | 3316.3 | 1463.8 | -192.6 | 1656.4 | -119.5 | 47.3 |  |  |  |  |  |
| 1H4S (AB:T) | 1HC7:AB |  | 2.85 | A | 2437.1 | 571.0 | 1.1 | 569.9 | 1.0 | 16.0 |  |  |  |  |  |
| 1J1U (AA':B) | 1U7D:A |  | 1.95 | A | 1943.1 | 695.6 | -144.8 | 840.5 | -160.9 | 32.7 |  |  |  |  |  |
| 1K8W (A:B) | 1R3F:A |  | 1.85 | D | 2088.7 | 367.0 | 103.3 | 263.7 | 112.5 | 21.2 |  |  |  |  |  |
| 1KQ2 (ABHIKM:R) | 1KQ1:ABHIKM |  | 2.71 | D | 3026.0 | -80.4 | -24.8 | -55.6 | -18.3 | -3.4 |  |  |  |  |  |
| 1M5O (C:B) | 1OIA:A |  | 2.2 | D | 1764.9 | 267.5 | -0.7 | 268.2 | -0.9 | 59.3 |  |  |  |  |  |
| 1M8V (AM:O) | 1H64:AM |  | 2.6 | D | 1289.3 | -103.8 | -222.2 | 118.4 | -368.7 | 13.6 |  |  |  |  |  |
| 1M8W (A:CE) | 1M8Z:A |  | 2.2 | D | 2142.2 | -72.1 | -84.8 | 12.7 | -87.1 | 0.8 |  |  |  |  |  |
| 1MSW (D:R) | 1ARO:P |  | 2.1 | C | 1479.3 | -1358.7 | -178.0 | -1180.8 | -243.2 | -31.2 |  |  |  |  |  |
| 1N35 (A:BC) | 1MUK:A |  | 2.5 | C | 3244.7 | 437.4 | -53.6 | 490.9 | -34.4 | 10.8 |  |  |  |  |  |
| 1N78 (A:C) | 1J09:A |  | 2.1 | A | 4510.6 | 272.4 | -124.7 | 397.2 | -58.8 | 20.5 |  |  |  |  |  |
| 1Q2R (AB:E) | 1R5Y:AC |  | 2.9 | C | 2378.7 | 588.5 | -101.1 | 689.6 | -87.6 | 27.1 |  |  |  |  |  |
| 1QF6 (A:B) | 1EVL:A |  | 2.9 | A | 3320.5 | -198.5 | -238.8 | 40.3 | -149.4 | 2.4 |  |  |  |  |  |
| 1SDS (C:FF') | 1XBI:A |  | 1.8 | B | 848.4 | -4.2 | -16.0 | 11.9 | -44.0 | 2.2 |  |  |  |  |  |
| 1SER (AB:T) | 1SES:AB |  | 2.9 | A | 2292.0 | -1299.0 | -140.1 | -1159.0 | -122.6 | -36.4 |  |  |  |  |  |
| 1U0B(B:A) | 1LI5:B |  | 2.3 | A | 3100.1 | -212.1 | -234.4 | 22.3 | -160.2 | 1.6 |  |  |  |  |  |
| 1UVI (A:D) | 1HHS:A |  | 2.15 | D | 1813.8 | 423.4 | 1.4 | 422.0 | 1.9 | 16.6 |  |  |  |  |  |
| 1VFG (B:D) | 4WBY:A |  | 2.8 | A | 1545.3 | -111.2 | -249.5 | 138.2 | -322.6 | 7.7 |  |  |  |  |  |
| 1WNE (A:BC) | 1U09:A |  | 3 | C | 3078.9 | 84.3 | -139.3 | 223.5 | -97.6 | 11.4 |  |  |  |  |  |
| 1WPU (A:C) | 1WPV:A |  | 1.48 | D | 1345.7 | -269.2 | -24.2 | -245.1 | -39.0 | -33.4 |  |  |  |  |  |
| 1YVP (B:EFH) | 1YVR:A |  | 2.2 | C | 3371.1 | 717.7 | 79.3 | 638.4 | 49.7 | 30.0 |  |  |  |  |  |
| 1ZBI (A:CD) | 1ZBF:A |  | 1.85 | C | 1631.2 | 201.5 | -51.7 | 253.2 | -67.2 | 40.7 |  |  |  |  |  |
| 2A8V (B:E) | 1A8V:B |  | 2.4 | D | 719.5 | 176.8 | -1.6 | 178.4 | -5.8 | 26.2 |  |  |  |  |  |
| 2ASB (A:B) | 1K0R:A |  | 1.5 | D | 2316.7 | 17.4 | 22.8 | -5.4 | 20.9 | -0.5 |  |  |  |  |  |
| 2AZ0 (AB:CD) | 2B9Z:AB |  | 2.6 | C | 2264.1 | -518.0 | -21.5 | -496.4 | -19.5 | -74.4 |  |  |  |  |  |
| 2AZX (AA':C) | 1R6U:AB |  | 2.8 | A | 2134.4 | 411.9 | -209.3 | 621.1 | -204.5 | 20.1 |  |  |  |  |  |
| 2BGG (A:PQ) | 1W9H:A |  | 2.2 | C | 2078.2 | 323.2 | -86.3 | 409.6 | -91.2 | 24.7 |  |  |  |  |  |
| 2EZ6 (AB:CD) | 1JFZ:AB |  | 2.05 |  | 1834.8 | -383.0 | -93.7 | -289.3 | -100.1 | -21.4 |  |  |  |  |  |
| 2F8S (AB:CD) | 1YVU:AD |  | 3 | C | 990.5 | -795.5 | -74.6 | -720.9 | -152.9 | -20.2 |  |  |  |  |  |
| 2FK6 (A:R) | 1Y44:A |  | 2.9 | C | 1134.7 | 3.1 | -38.0 | 41.1 | -65.4 | 3.4 |  |  |  |  |  |
| 2GIC (A:R) | 2QVJ:A |  | 2.92 | A | 2006.5 | -636.1 | -178.0 | -458.1 | -178.8 | -19.5 |  |  |  |  |  |
| 2GJW (AB:EFH) | 1R0V:AB |  | 2.85 | D | 3244.0 | 1313.2 | -626.5 | 1939.6 | -410.7 | 97.6 |  |  |  |  |  |
| 2GXB (A:EF) | 1QGP:A |  | 2.25 | C | 768.6 | -63.1 | 8.8 | -71.9 | 24.3 | -18.8 |  |  |  |  |  |
| 2HW8 (A:B) | 1AD2:A |  | 2.1 | C | 2107.1 | 710.3 | -2.3 | 712.6 | -2.2 | 68.0 |  |  |  |  |  |
| 2IX1 (A:B) | 2IX0:A |  | 2.74 | B | 4169.7 | 407.5 | -131.4 | 538.9 | -67.2 | 18.1 |  |  |  |  |  |
| 2JEA (AB:C) | 2JE6:AB |  | 2.33 | D | 1533.2 | 337.4 | 40.2 | 297.2 | 58.0 | 14.7 |  |  |  |  |  |
| 2JLU (A:C) | 2JLQ:A |  | 2.04 | D | 1925.7 | 184.9 | -525.1 | 710.1 | -620.5 | 35.1 |  |  |  |  |  |
| 2PY9 (B:E) | 2JZX:A |  | 2.56 | D | 1060.8 | 304.2 | -2.1 | 306.3 | -4.0 | 72.3 |  |  |  |  |  |
| 2Q66 (A:X) | 2HHP:A |  | 1.8 | D | 1795.7 | 178.3 | 163.6 | 14.7 | 198.7 | 0.7 |  |  |  |  |  |
| 2QUX (AB:C) | 2QUD:AB |  | 2.44 | D | 1753.8 | -141.1 | -92.8 | -48.3 | -115.9 | -4.3 |  |  |  |  |  |
| 2R7R (A:X) | 2R7Q:A |  | 2.6 | C | 1954.0 | -650.3 | -259.7 | -390.6 | -286.9 | -8.9 |  |  |  |  |  |
| 2XGJ (A:C) | 4U4C:A |  | 2.9 | D | 1389.4 | -544.7 | -262.5 | -282.2 | -409.2 | -6.8 |  |  |  |  |  |
| 2XNR (A:C) | 2XNQ:A |  | 1.6 | D | 926.0 | 12.5 | -91.4 | 104.0 | -226.1 | 24.0 |  |  |  |  |  |
| 2XZO (A:D) | 2XZP:A |  | 2.4 | D | 1972.3 | -1058.9 | -189.9 | -869.0 | -205.7 | -33.4 |  |  |  |  |  |
| 2Y8W (A:B) | 1WJ9:A |  | 1.8 | D | 2244.4 | -72.9 | -6.1 | -66.9 | -5.7 | -7.0 |  |  |  |  |  |
| 2ZKO (AB:CD) | 2Z0A:AB |  | 1.7 | C | 2458.2 | -412.5 | -156.6 | -255.8 | -126.8 | -36.4 |  |  |  |  |  |
| 3ADB (A:C) | 3A4M:A |  | 2.8 | C | 2469.5 | -2235.3 | -1066.1 | -1169.2 | -831.5 | -82.3 |  |  |  |  |  |
| 3AEV (B:C) | 2E3U:A |  | 2.8 | A | 2416.4 | -149.8 | -117.8 | -31.9 | -104.1 | -3.9 |  |  |  |  |  |
| 3AMT (A:B) | 3AU7:A |  | 2.9 | A | 2594.7 | -421.6 | -145.7 | -275.9 | -122.4 | -17.2 |  |  |  |  |  |
| 3BSN (A:PT) | 1SH3:A |  | 1.8 | D | 3111.9 | -75.1 | -269.1 | 194.0 | -185.8 | 9.5 |  |  |  |  |  |
| 3BX2 (A:C) | 3BWT:A |  | 2.84 | A | 2467.3 | -499.0 | -139.4 | -359.6 | -127.0 | -25.3 |  |  |  |  |  |
| 3D2S (A:E) | 3D2Q:A |  | 1.7 | C | 568.8 | -208.4 | -93.7 | -114.7 | -343.5 | -23.1 |  |  |  |  |  |
| 3DD2 (H:B) | 1JOU:AD |  | 1.9 | D | 1823.0 | 398.3 | -69.9 | 468.2 | -78.4 | 38.2 |  |  |  |  |  |
| 3EQT (AB:CD) | 2W4R:AD |  | 2 | D | 2285.1 | -541.1 | -263.9 | -277.2 | -254.1 | -20.4 |  |  |  |  |  |
| 3KS8 (AB:EF) | 3KS4:AB |  | 2.4 | C | 836.6 | -973.0 | -102.0 | -871.0 | -237.6 | -70.2 |  |  |  |  |  |
| 3FTE (A:CD) | 3FTD:A |  | 3.0 | C | 1646.9 | -65.5 | 152.0 | -217.5 | 192.4 | -17.6 |  |  |  |  |  |
| 3MDG (AB:C) | 3BAP:AC |  | 2.22 | C | 1069.0 | 258.8 | -31.2 | 289.9 | -61.9 | 15.0 |  |  |  |  |  |
| 3MOJ (B:A) | 2G0C:A |  | 2.9 | D | 1291.9 | -168.6 | -50.0 | -118.6 | -80.8 | -31.5 |  |  |  |  |  |
| 3NMR (A:B) | 2DHS:A |  | 1.85 | D | 1097.8 | 840.3 | -70.5 | 910.8 | -144.0 | 90.4 |  |  |  |  |  |
| 3O3I (X:A) | 2L5C:A |  | 2.8 | C | 923.0 | -830.8 | -85.5 | -745.3 | -211.2 | -119.1 |  |  |  |  |  |
| 3O8C (A:C) | 3O8B:A |  | 2 | D | 1909.3 | -218.3 | -192.6 | -25.7 | -211.8 | -1.0 |  |  |  |  |  |
| 3OIJ (AB:C) | 3OII:AB |  | 3.0 | C | 1786.5 | -419.0 | -46.5 | -372.5 | -56.4 | -20.5 |  |  |  |  |  |
| 3OL6 (A:BCD) | 1RA6:A |  | 2.5 | D | 4173.6 | 87.9 | -242.0 | 329.9 | -120.8 | 16.9 |  |  |  |  |  |
| 3PF4 (B:R) | 1CSP:A |  | 1.38 | D | 674.4 | -57.3 | 10.1 | -67.3 | 32.4 | -16.6 |  |  |  |  |  |
| 3QJJ (A:Q) | 3UFC:X |  | 2.8 | D | 3035.7 | -312.8 | -61.3 | -251.5 | -42.3 | -22.4 |  |  |  |  |  |
| 3RC8 (A:E) | 3RC3:A |  | 2.9 | C | 1473.0 | -713.3 | -36.6 | -676.7 | -54.1 | -23.2 |  |  |  |  |  |
| 3RW6 (A:H) | 3RW7:A |  | 2.3 | D | 2688.3 | -506.1 | -225.2 | -280.9 | -171.0 | -22.1 |  |  |  |  |  |
| 4E78 (A:PT) | 4E76:A |  | 2.9 | D | 1674.9 | -34.4 | -16.3 | -18.1 | -20.8 | -0.8 |  |  |  |  |  |
| 4ERD (AB:CD) | 4EYT:AB |  | 2.59 | C | 2248.9 | -526.6 | -286.4 | -240.2 | -277.5 | -19.6 |  |  |  |  |  |
| 4FVU (A:BC) | 3Q7C:A |  | 2.91 | C | 1248.1 | 113.2 | -3.9 | 117.1 | -6.7 | 11.2 |  |  |  |  |  |
| 4H5P (AB:E) | 3LYF:AB |  | 2.15 | D | 4482.8 | -3353.7 | -1023.2 | -2330.5 | -471.0 | -101.5 |  |  |  |  |  |
| 4HOR (A:X) | 4HOQ:A |  | 1.86 | D | 1672.3 | 646.4 | -30.1 | 676.5 | -41.4 | 29.5 |  |  |  |  |  |
| 4IG8 (A:BC) | 1PX5:A |  | 2.7 | C | 2417.1 | -457.9 | -195.5 | -262.5 | -160.4 | -18.1 |  |  |  |  |  |
| 4ILL (AB:RC) | 4ILR:AB |  | 2.48 | C | 7075.6 | -229.4 | -177.9 | -51.5 | -54.4 | -2.3 |  |  |  |  |  |
| 4J7M (A:B) | 4J7N:A |  | 1.5 | D | 1268.5 | -22.6 | -86.0 | 63.4 | -145.9 | 3.8 |  |  |  |  |  |
| 4M59 (AB:CD) | 4M57:AB |  | 2.46 | D | 7609.2 | -1973.0 | -1560.3 | -412.6 | -434.8 | -6.6 |  |  |  |  |  |
| 4MDX (AB:C) | 1NE8:AD |  | 1.5 | D | 2056.0 | 281.7 | 114.1 | 167.7 | 125.1 | 18.0 |  |  |  |  |  |
| 4N2Q (A:B) | 4ME2:A |  | 2.8 | D | 813.7 | -185.4 | -136.7 | -48.6 | -364.7 | -4.8 |  |  |  |  |  |
| 4QU6 (A:B) | 2LMI:A |  | 1.75 | D | 695.8 | 979.3 | 78.7 | 900.6 | 258.2 | 147.8 |  |  |  |  |  |
| 4YCP (A:B) | 4BFA:A |  | 2.55 | A | 2157.8 | -356.7 | -18.8 | -337.9 | -18.1 | -27.4 |  |  |  |  |  |
| 4YVJ (AB:C) | 3AXZ:AA' |  | 2.9 | A | 1204.4 | -97.5 | 32.9 | -130.3 | 57.5 | -9.4 |  |  |  |  |  |
| 4ZT0 (A:B) | 4CMP:A |  | 2.9 | C | 7866.4 | -2955.3 | -1405.9 | -1549.4 | -373.4 | -28.2 |  |  |  |  |  |
| 5BUD (A:B) | 5BTH:A |  | 1.99 | D | 1211.9 | -71.1 | -38.5 | -32.6 | -72.8 | -1.7 |  |  |  |  |  |
| 5DET (AB:PQ) | 5CYJ:AB |  | 1.95 | D | 2137.7 | 964.3 | -469.5 | 1433.8 | -523.9 | 155.8 |  |  |  |  |  |
| 5ED1 (A:BC) | 1ZY7:A |  | 2.77 | C | 1718.6 | -232.1 | -49.3 | -182.7 | -60.7 | -11.6 |  |  |  |  |  |
| 5EIM (A:C) | 5H8A:A |  | 1.54 | D | 1315.0 | -197.8 | 8.5 | -206.3 | 13.4 | -26.0 |  |  |  |  |  |
| 5ELS (AC:HI) | 5EL3:AB |  | 2.87 | D | 2112.2 | -556.9 | 153.8 | -710.7 | 158.2 | -52.5 |  |  |  |  |  |
| 5EX7 (A:B) | 4ZLR:A |  | 2.6 | D | 1639.7 | -23.8 | -71.2 | 47.4 | -97.5 | 4.4 |  |  |  |  |  |
| 5F5H (A:C) | 4QI0:A |  | 2.23 | C | 1618.4 | -14.6 | -86.6 | 72.0 | -109.8 | 10.0 |  |  |  |  |  |
| 5HR7 (A:D) | 3RFA:A |  | 2.4 | A | 3415.8 | -86.1 | -165.5 | 79.4 | -100.6 | 5.8 |  |  |  |  |  |
| **iii) P_B_R_U_ (10 Cases)** | | | | | | | | | | | | | | | |
| 1HQ1 (A:B) |  | 1DHU:A | 1.52 | C | 1364.6 |  |  |  |  |  | 2054.3 | 153.9 | 1900.4 | 227.4 | 262.7 |
| 1JID (A:B) |  | 1D4R:A | 1.8 | D | 1431.1 |  |  |  |  |  | 1040.1 | -74.3 | 1114.4 | -101.4 | 232.9 |
| 1LNG (A:B) |  | 1Z43:A | 2.3 | D | 2367.6 |  |  |  |  |  | -726.3 | -177.4 | -548.9 | -145.1 | -36.3 |
| 2I82 (A:E) |  | 3L0U:A | 2.05 | D | 2816.2 |  |  |  |  |  | -160.5 | -326.0 | 165.5 | -224.4 | 72.4 |
| 2OZB (AB:C) |  | 2XEB:AB | 2.6 | C | 2204.9 |  |  |  |  |  | 267.9 | -114.6 | 382.6 | -100.2 | 82.8 |
| 2PJP (A:B) |  | 1MFK:A | 2.3 | C | 1300.3 |  |  |  |  |  | 9.2 | -159.4 | 168.6 | -237.0 | 43.8 |
| 2ZM5 (A:C) |  | 3L0U:A | 2.55 | A | 3701.5 |  |  |  |  |  | -178.9 | -204.1 | 25.3 | -108.8 | 13.4 |
| 3BT7 (A:C) |  | 1EVV:A | 2.43 | C | 2230.2 |  |  |  |  |  | -309.2 | -310.4 | 1.2 | -248.6 | 0.5 |
| 3IEV (A:D) |  | 1SDR:B | 1.9 | D | 2273.2 |  |  |  |  |  | -352.1 | -311.7 | -40.5 | -247.4 | -24.1 |
| 3ZJT (A:B) |  | 2NQP:F | 2.2 | A | 634.3 |  |  |  |  |  | 319.1 | -28.1 | 347.2 | -85.1 | 56.0 |

^a^Four letter PDB code of the protein–protein complexes and their corresponding unbound form of interacting subunits. For the bound structures Chain identifiers of the interacting subunits molecules are mentioned in the parenthesis separated by colon. For unbound structures the chain identifiers are provided along with the PDB code separated by colon.

All the parameters (along with their abbreviations) are discussed in the main article.

## **Table S2.** Change in accessibility upon binding

| Bound  Unbound | Interface (Å^2^) | | | Non-interface (Å^2^) | | |
| --- | --- | --- | --- | --- | --- | --- |
|  | Helix | Sheet | Loop | Helix | Sheet | Loop |
| Helix | –3.3 | 0.0 | –2.5 | –0.3 | 0.0 | –3.1 |
| Sheet | 0.0 | –0.9 | –1.1 | 4.7 | 0.1 | 1.9 |
| Loop | –1.5 | 7.7 | –1.2 | 0.0 | 1.3 | –0.2 |

Average change in ∆A per residue transition of different secondary structural elements upon binding.
